# Baseline resistance to nucleoside reverse transcriptase inhibitors fails to predict virologic response to combination therapy in children (PACTG 338)

**DOI:** 10.1186/1742-6405-4-2

**Published:** 2007-02-06

**Authors:** Susan A Fiscus, Andrea Kovacs, Leslie A Petch, Chengcheng Hu, Andrew A Wiznia, Lynne M Mofenson, Ram Yogev, Kenneth McIntosh, Stephen I Pelton, Sonia Napravnik, Kenneth Stanley, Sharon A Nachman

**Affiliations:** 1 Department of Microbiology and Immunology, University of North Carolina School of Medicine, Chapel Hill, NC, USA; 2 Center for AIDS Research, University of North Carolina, Chapel Hill, NC, USA; 3 Maternal, Child and Adolescent Program, University of Southern California Medical Center, Los Angeles, CA, USA; 4 Center for Biostatistics in AIDS Research and Department of Biostatistics, Harvard School of Public Health, Boston, MA, USA; 5 Department of Pediatrics, Jacobi Medical Center and Albert Einstein College of Medicine, Bronx, NY, USA; 6 Pediatric, Adolescent and Maternal AIDS Branch, National Institute of Child Health and Human Development, National Institutes of Health, Rockville, MD, USA; 7 Division of Infectious Diseases, Children's Memorial Hospital and Northwestern University School of Medicine, Chicago, IL, USA; 8 Division of Infectious Diseases, Children's Hospital and Harvard Medical School, Boston, MA, USA; 9 Department of Pediatrics, Boston Medical Center, Boston, MA, USA; 10 Department of Pediatrics, SUNY Health Science Center at Stony Brook, Stony Brook, NY, USA

## Abstract

**Background:**

The association between baseline drug resistance mutations and subsequent increase in viral failure has not been established for HIV-infected children. We evaluated drug resistance mutations at 39 codon sites (21 protease inhibitor (PI) resistant codons and 18 nucleoside reverse transcriptase inhibitor (NRTI) resistant codons) for 92 clinically stable NRTI-experienced, PI-naive HIV-infected children 2 to 17 years of age who were initiating new therapy with ritonavir plus zidovudine (ZDV) and lamivudine or plus stavudine. The association between baseline drug resistance mutations and subsequent viral failure after 12 and 24 weeks of highly active antiretroviral therapy (HAART) was studied.

**Results:**

There were few primary PI associated mutations in this PI-naïve population, but 84% had NRTI mutations – codons 215 (66%), 41 (42%), 67 (37%), 210 (33%) and 70 (32%). None of the specific baseline drug resistance mutations were associated with a higher rate of virologic failure after 12 or 24 weeks of HAART. Median week 12 viral load decreased as the total number of NRTI mutations at baseline increased (P = 0.006). Specifically, a higher level of baseline ZDV resistance mutation was associated with a decrease in viral failure after 12 weeks on a ZDV-containing HAART regimen (P = 0.017).

**Conclusion:**

No increase was seen in the rate of viral failure after HAART associated with the presence of resistance mutations at baseline. This paradoxical result may be due to adherence, replicative capacity, or ZDV hypersusceptibility to the new regimen.

## Background

Nucleoside reverse transcriptase inhibitors (NRTI) were the first antiretroviral drugs available and continue to be a component of anti-retroviral therapy (ART), despite the emergence of drug resistance over time. Few studies have investigated the role of pre-existing drug resistance and response to therapy in children [1-4] compared to similar studies in adults [5,6]. The largest published drug resistance study of HIV-infected children found a high rate of primary mutations associated with resistance to zidovudine (ZDV), didanosine (ddI) and zalcitabine (ddC), but concluded that none of the baseline drug mutations were associated with a higher rate of virologic failure [2]. It is possible that HIV drug resistance may evolve differently in children because of differences in pharmacokinetics in children, fewer drug options, and higher viral burden, especially in younger children [7,8] and unique challenges to therapy compliance. Pediatric AIDS Clinical Trials Group (PACTG) 338 was one of the first clinical trials to evaluate highly active anti-retroviral therapy (HAART) which included a protease inhibitor, ritonavir (RTV), in children [9]. We investigated the role of baseline HIV drug resistance mutations and response to therapy.

## Results

There were very few primary resistance mutations to PIs in this PI-naïve population, although 88% of the children had polymorphisms that included secondary minor resistance mutations. The most frequent secondary PI mutations were at codons 63 (78%), 77 (37%), 36 (17%) and 10 (12%) (data not shown). Only two children had a primary PI resistance mutation (V82A). Other PI mutations (71, 33 and 20) were present in less than 10% of the study subjects. The most common NRTI mutations occurred at codons 215 (66%), 41 (42%), 67 (37%), 210 (33%), 70 (32%), 69 (22%), 118 (21%) and 219 (21%). The median numbers of baseline NRTI, thymidine analog mutations (TAM), PI and total mutations were 3, 3, 2 and 4.5, respectively (both primary and secondary mutations were included in the analysis for the PI mutations).

After 12 weeks on study, 51 (55%) subjects had viral loads suppressed below 400 copies/ml. The number of subjects with viral suppression dropped to 31 (34%) and 29 (32%) at weeks 24 and 48, respectively.

The association between the presence of a specific baseline mutation and virologic failure after 12 weeks of HAART was studied (Table 1). There was the suggestion of a potential association with virologic failure for only one baseline mutation, the NRTI codon 215 (unadjusted P = 0.019) for the three-drug combination regimen. However, in this case the presence of resistance mutations was associated with a decreased (rather than an increased) rate of viral failure at week 12.

**Table 1:** Association of baseline NTRI resistance mutations and viral failure after 12 weeks on HAART

	d4T plus RTV group Number with a mutation		ZDV plus 3TC plus RTV group Number with a mutation		
		
Baseline resistance mutation codons	RNA > 400 at week 12 (N = 19)	RNA ≤ 400 at week 12 (N = 26)	RNA > 400 at week 12 (N = 22)	RNA ≤ 400 at week 12 (N = 25)	Total number (%) with a resistance mutation at baseline (N = 92 children)
NRTI resistance mutations^a^					

215	14	19	9^b^	19^b^	61 (66)
41	8	11	6	14	39 (42)
67	4	13	6	11	34 (37)
210	4	12	4	10	30 (33)
70	4	10	6	9	29 (32)
69	4	4	5	7	20 (22)
118	2	7	5	5	19 (21)
219	3	8	3	5	19 (21)
74	2	6	1	4	13 (14)
184	2	1	1	1	5 (5)
44	0	2	0	2	4 (4)
151	0	0	0	2	2 (2)
65	0	1	0	0	1 (1)
75	0	1	0	0	1 (1)
116	0	0	0	1	1 (1)
Any NRTI	16	23	15	23	77 (84)
Any PI or NRTI	19	25	21	25	90 (98)

a. No cases with mutations were observed for the following NRTI mutations: 62, 77 and 115.

b. P = 0.019, Fisher's exact test

**Figure 1:**
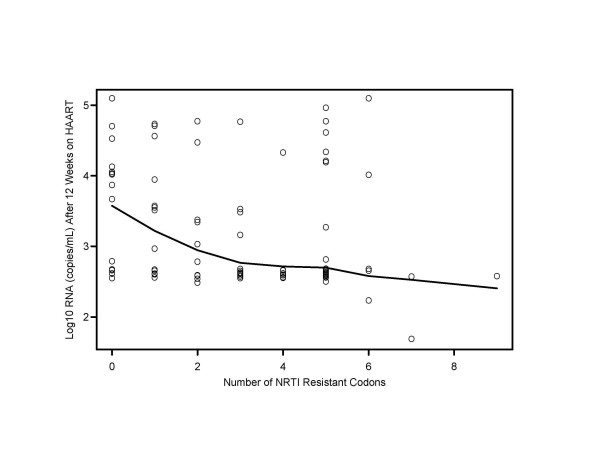
Association between the number of baseline nucleoside reverse transcriptase inhibitor mutations and viral load after 12 weeks on HAART (Viral load is the log_10 _RNA copies/mL value at 12 weeks.)

When the association between the number of baseline resistance mutations and virologic failure after 12 weeks of HAART was investigated, the only significant relationship observed was in the ZDV/lamivudine (3TC)/RTV combination regimen. A higher median number of NRTI mutations at baseline was associated with virologic suppression (1.5 versus 4.0, P = 0.016). There was no apparent association between the number of baseline PI resistance mutations and the level of viral load after 12 weeks of HAART, although as the number of baseline NRTI mutations increased the level of viral load at week 12 decreased (Figure 1). A linear regression with adjustment for censoring of viral loads at the detection limit showed that after 12 weeks on HAART there was a decrease of 0.245 in log_10 _RNA (copies/mL) associated with each additional NRTI resistance mutation (P = 0.006).

Association between the level of baseline ZDV resistance mutations and viral failure after 12 weeks on HAART was investigated (Table 2). The level of baseline ZDV resistance was categorized as i) high level (NRTI codon 215), ii) intermediate level (at least one of the NRTI codons 41 or 210, but no high level mutation), iii) low level (at least one of the NRTI codons 67, 70 or 219, but no high or intermediate level mutation), or iv) no ZDV resistance according to an algorithm specified prior to any data analysis. There was no association between baseline ZDV resistance levels and viral failure after 12 weeks on HAART for the two-drug HAART arm, although for the ZDV-containing three-drug arm a higher level of baseline ZDV resistance was associated with a lower chance of viral failure (P = 0.017). From Table 2 it should be noted that whenever a codon 41 or 210 mutation was observed a codon 215 mutation was also observed and the case was classified as having a high level of resistance. Thus the association between a high level of resistance and a lower chance of viral failure should not be ascribed to only the occurrence of a codon 215 mutation, but rather to the occurrence of codon 215, 41 and/or 210 mutations.

**Table 2:** Association of the level of baseline ZDV resistance mutations and viral failure after 12 weeks on HAART

	d4T plus RTV group		ZDV plus 3TC plus RTV group	
Level of baseline ZDV resistance mutation	RNA > 400 at week 12 (N = 19)	RNA ≤ 400 at week 12 (N = 26)	RNA > 400 at week 12 (N = 22)	RNA ≤ 400 at week 12 (N = 25)

High^a^	14	19	9	19
Intermediate^b^	0	0	0	0
Low^c^	1	3	4	2
None	4	4	9	4
P-value^d^	0.904		0.017	

a. At least codon 215.

b. At least one of codons 41 and 210, but no high level mutation (none at codon 215).

c. At least one of codons 67, 70 and 219, but no high or intermediate level mutations (none at codons 215, 41 or 210).

d. Mantel-Haenszel test for trend.

The association between the presence of a specific baseline mutation and virologic suppression during the first 24 weeks on HAART was explored. Viral response was categorized into three ordered levels: full suppression (viral load no more than 400 copies/mL at week 12 or 24), partial suppression (viral load dropped 0.75 log_10 _copies/mL or more at week 12 or 24 from baseline, but no full suppression), and no suppression. According to this definition, 56 (60%) subjects achieved full viral suppression and an additional 13 (14%) achieved partial viral suppression during the first 24 weeks on HAART. There was no statistically significant (at the unadjusted 5% level) association between any baseline mutation and virologic suppression during the first 24 weeks on HAART.

For the 69 children with full or partial viral suppression as defined above, the association between the presence of a specific baseline mutation and viral rebound between the suppression and week 48 was evaluated. A rebound was declared if there was an increase in RNA of more than one log_10 _at any time between the time of suppression and week 48 from the nadir viral load value at or before the time of suppression, or if the child came off the assigned protocol treatment at or before week 48. Of the 69 children who achieved full or partial virologic suppression during the first 24 weeks on HAART, 37 (54%) had a viral rebound. In the three-drug arm children with mutation at the NRTI codon 67 seemed to be less likely to have a viral rebound after virologic suppression (unadjusted P = 0.002). There was also the suggestion of a potential association with virologic failure for the presence of any PI mutation (unadjusted P = 0.020) in the three-drug arm and for mutation at the NRTI codon 69 (unadjusted P = 0.028) in the two-drug arm, but these findings need to be treated cautiously due to the problem of multiple comparisons (see statistical analysis section).

## Discussion

We evaluated the association between resistance mutations and their potential effect on the ability of antiviral medications to reduce the viral burden in children with HIV disease. As expected, there was little PI resistance and considerable NRTI resistance in this PI-naïve, NRTI-experienced population. However, contrary to our expectations, we did not find an increase in the rate of viral failure after HAART associated with the presence of resistance mutations at baseline. The two specific associations identified at week 12 in our study (Table 1) were of borderline significance and were in the direction of a decrease, rather than an increase, in the rate of viral failure. As the number of NRTI mutations at baseline increased, the median viral load at week 12 decreased (P = 0.006) and as the baseline level of ZDV resistance increased viral failure decreased after 12 weeks on a ZDV-containing HAART regimen (P = 0.017). Our results were consistent with the drug resistance study of 135 HIV-1 infected children by Eschleman et al. [2]. Neither study produced significant evidence linking a specific baseline drug resistance mutation to a subsequent increase in viral failure. These results suggest that baseline genotyping may not be necessary prior to switching to a PI based regimen. This finding has particular pertinence in resource limited settings.

Possible explanations for these observations include adherence, replicative capacity, and hypersusceptibility to NRTIs. It is likely that children who were adherent to a non-suppressive NRTI regimen and thus developed resistance, were more likely to be adherent to a HAART regimen that contained a brand new class of drugs. Friedland and Williams [10] have suggested that the relationship between the development of resistance and adherence to the drugs is bell-shaped, such that both high and low degrees of adherence decrease the likelihood of the development of resistance. This concept has been supported by several recent studies [11-13]. The RNA results from PACTG 338, where most children had detectable, even high viral loads at baseline, suggest that the drug(s) they were taking were less effective in suppressing viral replication, resulting in ongoing viral production in the presence of ART. Thus, children who were more adherent may have been more likely to develop resistance to their non-suppressive initial therapy regimens The children's viral loads rapidly became undetectable once they were placed on drugs that were more suppressive and to which they had no baseline resistance (RTV and 3TC). Only 5 of the children had the M184V and only 2 had the V82A mutations at baseline. The effect would be less striking in children in the two drug regimen since the mutations associated with stavudine (d4T) resistance are primarily those seen with ZDV, ddI and ddC to which the children had been previously exposed (codons 41, 67, 70, 210, 215, and 219).

Another explanation for these results might be reduced replication capacity of the virus found in the children at baseline. The NRTI mutations typically associated with reduced fitness are M184V [14] and K65R [15]; however, only 1 child had the K65R mutation and only 5 had the M184V mutation at baseline. In addition, viral loads at baseline were relatively high: the median baseline viral load was 20,500 copies/mL and 20% of the children had baseline viral loads of 100,000–1,000,000 copies/mL.

Lastly, it has been shown that the L74V and M184V mutations confer hypersusceptibility to ZDV [16,17]. However, in the 338 data set only 5 of the 47 children randomized to ZDV/3TC/RTV for whom we have data, had the 74V mutation. This could, however, be an explanation for why children on the ZDV/3TC/RTV arm who had more baseline NRTI mutations were more likely to have a viral load <400 copies/mL at 12 weeks than those with fewer mutations.

Limitations of our study include the fact that we have baseline resistance data on only a subset of children from the entire study and lack of information on adherence to the drug regimens, replicative capacity and hypersusceptibility of the viruses. We did not determine the occurrence of new resistance mutations after the initiation of HAART or investigate the virologic impact of these subsequent resistance mutations.

## Conclusion

Despite considerable baseline NRTI resistance, the children in PACTG 338 who were treated with a brand new class of drugs (e.g. ritonavir, the protease inhibitor) in combination therapy responded favorably and rapidly. We did not observe an increase in the rate of viral failure after HAART linked to the presence of resistance mutations at baseline. In fact, viral loads at the 12 week time point were inversely correlated with the number of baseline NRTI mutations. It is important to remember that resistance is not an all-or-none phenomenon, and treatment failure is not defined by a resistance test. In order to better understand both the virology of mutated viruses in vivo and their response to anti-retroviral drugs as well as the use of resistance testing, prospective randomized clinical trials linked with pathogenesis-related in vitro analysis should be performed in children.

## Methods

### Patient population

PACTG 338 was a multicenter, randomized clinical trial that enrolled 297 children aged 2–17 years. All subjects were HIV-infected, had been receiving continuous, unchanged antiretroviral therapy for the 16 weeks before study entry, and were naïve to protease inhibitors and lamivudine (3TC) or had received no more than 6 weeks of ZDV plus 3TC in the year prior to study entry and none in the 4 months prior to study entry. Children were randomly assigned to receive either ZDV/3TC (n = 100), stavudine (d4T)/RTV (n = 97) or ZDV/3TC/RTV (n = 100). Children initially assigned to the ZDV/3TC regimen were not included in this evaluation of drug resistance and subsequent viral failure as this regimen was demonstrated to result in a suboptimal virologic suppression compared to protease inhibitor-based regimens.

A total of 92 children assigned to a RTV-containing regimen had both baseline resistance information and viral load measurement at week 12, and they were included in any analysis involving the primary endpoint of viral failure at week 12. Of these 92 subjects, the median age was 7.3 years, median CD4 cell count was 602 cells/mm^3^, proportion with CD4 cell count <500 cells/uL was 29%, proportion with CD4 percent <25% was 38%, median plasma HIV-1 RNA was 20,500 copies/mL, and the proportion with HIV-1 RNA copy number > 4 log_10 _was 75%. The majority of the children were African-American (66%) and 51% were male. Children had received prior treatment with ZDV monotherapy (46%), the combination of ZDV and ddI (37%) or other combination therapy (15%). Baseline characteristics of the children in this resistance analysis were very similar to the baseline characteristics for the overall PACTG 338 study of 297 children, except for minor differences in the CD4 cell count (648 cells/mm^3^), the proportion of African-American children (51%), and the proportion with HIV-1 RNA copy number > 4 log_10 _(64%). Children with viral loads <1,000 copies/mL were excluded from this resistance study. For the secondary analyses of viral suppression at weeks 12 or 24 and viral rebound after 48 weeks on HAART, two additional subjects were included, who did not have viral load measured at week 12 but did have information at week 24. The institutional review board at each institution approved the study and informed consent was obtained from all patients or their guardians.

### HIV-1 genotyping

Sequencing was determined in batch at the conclusion of the study in two laboratories that participated in the National Institute of Allergy and Infectious Diseases (NIAID) Virology Quality Assurance Program. For HIV-1 sequencing, plasma RNA was extracted using the QIAampViral RNA Mini Kit (Qiagen Inc., Valencia, CA). Reverse transcriptase polymerase chain reaction (RT-PCR) and direct DNA sequencing of protease and reverse transcriptase genes were performed using the TruGene HIV-1 Genotyping Kit (Visible Genetics-Bayer Diagnostics, Toronto, Canada) according to the manufacturer's instructions. The International AIDS Society-USA recommended guidelines for resistance to protease inhibitor (PI) and nucleoside reverse transcriptase inhibitors (NRTI) were used [18,19].

### Viral load

HIV-1 RNA copy number was assessed using the NucliSens Assay (Organon Teknika, Durham, NC) [20], which has a lower limit of quantification of 400 copies/mL. All RNA assays were performed at a single laboratory at the University of North Carolina, Chapel Hill, NC that was certified as proficient by the NIAID Virology Quality Assurance Program [21]. Assay results from the NucliSens Assay were adjusted using Virology Quality Assurance external standards [22].

### Statistical analysis

Fisher's exact test was used for associations between specific baseline resistance mutations and viral failure, the Kruskal-Wallis test was used to assess the association between the number of resistance mutations and viral failure, and the Mantel-Haenszel test for trend was used to evaluate the association between level of baseline ZDV resistance and viral failure defined as viral load > 400 cp/ml [23,24]. Further, the Kruskal-Wallis test was used to correlate specific baseline resistance mutations and viral suppression, Fisher's exact test was used to investigate the association of baseline mutations and viral rebound, and the association between the total number of resistance mutations and viral load was assessed using the locally weighted scatter plot smooth [25] and linear regression with adjustment for left censoring. All P values were two-sided and were not adjusted for multiple comparisons. Because 39 codon sites were evaluated in this analysis, caution should be exercised in the interpretation of the P values. A conservative solution to the multiple comparisons problem is the Bonferroni method that multiplies the nominal P value times the overall number of statistical tests [26]. If the result is still <0.05, then the result is clearly statistically significant. Using the Bonferroni approach for any analysis involving individual resistance codons, a P value between 0.0013 (0.05/39) and 0.05 should be interpreted as suggestive but not necessarily definitive. P < 0.0013 should be considered clear evidence of statistical significance.

## Competing interests

The author(s) declare that they have no competing interests.

## Authors' contributions

AAW, LMF, RY, KM, SIP, KES and SAN conceived, designed and implemented the primary study (PACTG 338), upon which this secondary resistance study was based. SF organized this secondary study and contributed to its design and implementation along with SAF, AK, LAP and SN. CH performed the statistical analysis. SF drafted the manuscript jointly with KES. All authors provided review comments and textual modifications during manuscript development. All authors read and approved the final manuscript.

## References

[B1] Principi N, Marchisio P, Esposito S, Rossi P, Gattinara GC, Galli L, Gabiano C, Zuccotti GV, Orlandi P (1998). Zidovudine therapy and HIV type 1 mutations in children with symptomatic HIV type 1 infection: effect of switching to didanosine or zidovudine plus didanosine therapy. AIDS Res Hum Retroviruses.

[B2] Eshleman SH, Krogstad P, Jackson JB, Wang YG, Lee S, Wei LJ, Cunningham S, Wantman M, Wiznia A, Johnson G, Nachman S, Palumbo P (2001). Analysis of human immunodeficiency virus type 1 drug resistance in children receiving nucleoside analogue reverse-transcriptase inhibitors plus nevirapine, nelfinavir, or ritonavir (Pediatric AIDS Clinical Trials Group 377). J Infect Dis.

[B3] Perez EE, Rose SL, Peyser B, Lamers SL, Burkhardt B, Dunn BM, Hutson AD, Sleasman JW, Goodenow MM (2001). Human immunodeficiency virus type 1 protease genotype predicts immune and viral responses to combination therapy with protease inhibitors (PIs) in PI-naïve patients. J Infect Dis.

[B4] Mullen J, Leech S, O'Shea S, Chrystie IL, du Mont G, Ball C, Sharland M, Cottam F, Zuckerman M, Rice P, Easterbrook P (2002). Antiretroviral drug resistance among HIV-1 infected children failing treatment. J Med Virol.

[B5] Durant J, Clevenbergh P, Halfon P, Delgiudice P, Porsin S, Simonet P, Montagne N, Boucher CAB, Schapiro JM, Dellamonica P (1999). Drug-resistance genotyping in HIV-1 therapy: the VIRAD APT randomised controlled trial. Lancet.

[B6] Shafer RW (2002). Genotypic testing for human immunodeficiency virus type 1 drug resistance. Clin Microbiol Rev.

[B7] Shearer WT, Quinn TC, LaRussa P, Lew JF, Mofenson L, Almy S, Rich K, Handelsman E, Diaz C, Pagano M, Smeriglio V, Kalish LA (1997). Viral load and disease progression in infants infected with human immunodeficiency virus type 1. N Engl J Med.

[B8] Mofenson LM, Korelitz J, Meyer WA, Bethel J, Rich K, Pahwa S, Moye J, Nugent R, Read J (1997). The relationship between serum human immunodeficiency virus type 1 (HIV-1) RNA level, CD4 lymphocyte percent, and long-term mortality risk in HIV-1-infected children. J Infect Dis.

[B9] Nachman SA, Stanley K, Yogev R, Pelton S, Wiznia A, Lee S, Mofenson L, Fiscus S, Rathore M, Jimenez E, Borkowsky W, Pitt J, Smith ME, Wells B, McIntosh K (2000). Nucleoside analogs plus ritonavir in stable antiretroviral therapy-experienced HIV-infected children: a randomized controlled trial. JAMA.

[B10] Friedland GH, Williams A (1999). Attaining higher goals in HIV treatment: the central importance of adherence. AIDS.

[B11] Bangsberg DR, Charlebois ED, Grant RM, Holodniy M, Deeks SG, Perry S, Conroy KN, Clark R, Guzman D, Zolopa A, Moss A (2003). High levels of adherence do not prevent accumulation of HIV drug resistance mutations. AIDS.

[B12] Walsh JC, Pozniak AL, Nelson MR, Mandalia S, Gazzard BG (2002). Virologic rebound on HAART in the context of low treatment adherence is associated with a low prevalence of antiretroviral drug resistance. JAIDS.

[B13] Sethi AK, Celentano DD, Gange SJ, Moore RD, Gallant JE (2003). Association between adherence to antiretroviral therapy and human immunodeficiency virus drug resistance. Clin Infect Dis.

[B14] Van Rompay KKA, Matthews TB, Higgins J, Canfield DR, Tarara RP, Wainberg MA, Schinazi RF, Pedersen NC, North TW (2002). Virulence and reduced fitness of simian immunodeficiency virus with the M184V mutation in reverse transcriptase. J Virol.

[B15] Weber J, Chakraborty B, Weberova J, Miller MD, Quinones-Mateu ME (2005). Diminished replicative fitness of primary human immunodeficiency virus type 1 isolates harboring the K65R mutation. J Clin Microbiol.

[B16] Tisdale M, Kemp SD, Parry NR, Larder BA (1993). Rapid in vitro selection of human immunodeficiency virus type 1 resistant to 3'thiacytodine inhibitors due to a mutation in the YMDD region of reverse transcriptase. Proc Natl Acad Sci USA.

[B17] Miranda LR, Gotte M, Liang F, Kuritzkes DR (2005). The L74V mutation in human immunodeficiency virus type 1 reverse transcriptase counteracts enhanced excision of zidovudine monophosphate associated with thymidine analog resistance mutations. Antimicrob Agents Chemother.

[B18] Johnson VA, Brun-Vezinet F, Clotet B, Conway B, D'Aquila RT, Demeter LM, Kuritzkes DR, Pillay D, Schapiro JM, Telenti A, Richman DD (2004). Update of the drug resistance mutations in HIV-1:2004. Top HIV Med.

[B19] Yeni PG, Hammer SM, Carpenter CCJ, Cooper DA, Fischl MA, Gatell JM, Gazzard BG, Hirsch MS, Jacobsen DM, Katzenstein DA, Montaner JSG, Richman DD, Saag MS, Schechter M, Schooley RT, Thompson MA, Vella S, Volberding PA (2002). Antiretroviral treatment for adult HIV infection in 2002: updated recommendations of the International AIDS Society-USA Panel. JAMA.

[B20] Dyer JR, Pilcher CD, Shepard R, Schock J, Eron JJ, Fiscus SA (1999). Comparison of NucliSens and Roche Monitor assays for quantitation of levels of human immunodeficiency virus type 1 RNA in plasma. J Clin Microbiol.

[B21] Yen-Lieberman B, Brambilla D, Jackson B, Bremer J, Coombs R, Cronin M, Herman S, Katzenstein D, Leung S, Lin HJ, Palumbo P, Rasheed S, Todd J, Vahey M, Reichelderfer P (1996). Evaluation of a quality assurance program for quantitation of human immunodeficiency virus type 1 RNA in plasma by the AIDS Clinical Trials Group virology laboratories. J Clin Microbiol.

[B22] Brambilla D, Leung S, Lew J, Todd J, Herman S, Cronin M, Shapiro DE, Bremer J, Hanson C, Hillyer GV, McSherry GD, Sperling RS, Coombs RW, Reichelderfer PS (1998). Absolute copy number and relative change in determinations of human immunodeficiency virus type 1 RNA in plasma: effect of an external standard on kit comparisons. J Clin Microbiol.

[B23] Hollander M, Wolfe DA (1999). Nonparametric Statistical Methods.

[B24] Mantel N (1963). Chi-square tests with one degree of freedom: extensions of the Mantel-Haenszel procedure. J Am Stat Assoc.

[B25] Cleveland WS (1979). Robust locally weighted regression and smoothing scatterplots. J Am Stat Assoc.

[B26] Pocock SJ (1997). Clinical trials with multiple outcomes: a statistical perspective on their design, analysis, and interpretation. Controlled Clin Trials.

